# Response to: ‘Failed oral immunotherapy should be considered as a risk factor for fatal anaphylaxis, and omalizumab treatment considered’

**DOI:** 10.1186/s13052-024-01815-6

**Published:** 2024-11-10

**Authors:** Elio Novembre, Mariannita Gelsomino, Lucia Liotti, Simona Barni, Francesca Mori, Mattia Giovannini, Carla Mastrorilli, Luca Pecoraro, Francesca Saretta, Riccardo Castagnoli, Stefania Arasi, Lucia Caminiti, Angela Klain, Michele Miraglia del Giudice

**Affiliations:** 1 Department of Health Sciences, University of Florence, Florence, 50139 Italy; 2 Department of Life Sciences and Public Health, Pediatric Allergy Unit, University Foundation Policlinico Gemelli IRCCS Catholic University of the Sacred Heart, Rome, Italy; 3 Pediatric Unit, Department of Mother and Child Health, Salesi Children’s Hospital, Ancona, 60123 Italy; 4 Allergy Unit, Meyer Children’s Hospital IRCCS, Florence, 50139 Italy; 5 Pediatric Hospital Giovanni XXIII, Pediatric and Emergency Department, AOU Policlinic of Bari, Bari, 70126 Italy; 6 Pediatric Unit, Department of Surgical Sciences, Dentistry, Gynecology and Pediatrics, University of Verona, Verona, 37126 Italy; 7 Pediatric Department, Latisana-Palmanova Hospital, Azienda Sanitaria Universitaria Friuli Centrale, Udine, 33100 Italy; 8 Department of Clinical, Surgical, Diagnostic and Pediatric Sciences, University of Pavia, Pavia, 27100 Italy; 9 Pediatric Clinic, Fondazione IRCCS Policlinico San Matteo, Pavia, 27100 Italy; 10 Translational Research in Pediatric Specialties Area, Division of Allergy, Bambino Gesù Children’s Hospital, IRCCS, Rome, 00165 Italy; 11 Allergy Unit, Department of Pediatrics, AOU Policlinico Gaetano Martino, Messina, 98124 Italy; 12 Department of Woman, Child and General and Specialized Surgery, University of Campania “Luigi Vanvitelli”, Naples, 80138 Italy

**Keywords:** Fatal anaphylaxis, Food allergy, Oral immunotherapy, Epinephrine

Dear Editor,

We thank Nicolardi et al. [1] for appreciating our paper and for mentioning a possible overlooked issue regarding the potential risk for severe reactions in patients who have not successfully completed Oral Immunotherapy (OIT) and may encounter the allergen unintentionally. This topic is of great interest in the OIT area.

In the paper of Novembre et al. [2], this aspect was generally mentioned in the discussion: “Therefore, in children with persistent milk allergy (CMA) and OIT failure, particular psychological and practical support is needed”. No other specific suggestion could be made, as no studies clearly demonstrated an association between the stop of OIT and an increased risk of severe reactions.

In particular, in the retrospective analysis of Badina et al. [3] cited in the letter of a cohort of 342 children affected by persistent CMA who started OIT, the reported risk of life-threatening events among patients assuming milk during OIT than in those who stopped the protocol was not statistically significant (3.5% vs. 6.3%).

Moreover, the same study [3], as reported by the Authors in the discussion, has significant limits: “First, a significant percentage of patients lost at follow-up in 20 years. Second, the reactions were not classified in a standardized way due to the retrospective character of the study. Furthermore, the reactions’ recording was based on family histories, subjectively experienced, and often distant in time. Reactions were graded to reduce this recall bias according to their frequency and the main symptom of the most severe ones. Finally, since this was a single‐center study, the sample size was limited and possibly not big enough to allow statistically significant conclusions”.

In conclusion, the Authors themselves state: “So far, the data from our study are insufficient to demonstrate a statistically significant correlation between OIT discontinuation and a higher risk of severe reactions, and larger cohorts may be needed to confirm these findings.”

For all these reasons, an increased risk of life-threatening reactions among patients who stopped OIT was not reported in the study of Novembre et al. [2].

Regarding the use of omalizumab in patients who previously failed milk OIT, which was not the focus of the work of Novembre et al. [2], the cited study by Badina et al. [4] considered four patients. Moreover, of these four patients, “After 2 months of the Omalizumab (OML) discontinuation, one patient (Patient 2) resumed OML, because of deterioration in control of asthma. Due to a high level of fear and anxiety, one subject (Patient 3) chose to drastically reduce the cow milk (CM) maintenance dose after OML withdrawal even without any reaction”. The Authors themselves conclude that “Due to the limits of this study (small sample size and the follow-up still in progress), far more data are needed to establish the safety and effectiveness of this approach” and we agree with this conclusion.

## Data Availability

Not applicable.

## Abbreviations

OIT: Oral Immunotherapy
CMA: Cow’s milk allergy
OML: Omalizumab
CM: Cow milk
